# Feeling Like a Woman: Interoception and the Objectified Body

**DOI:** 10.3390/brainsci16050494

**Published:** 2026-04-30

**Authors:** Tomi-Ann Roberts, James W. Pennebaker, Benita Jackson

**Affiliations:** 1 Department of Psychology and Neuroscience, Colorado College, Colorado Springs, CO 80903, USA; 2 Department of Psychology, University of Texas at Austin, Austin, TX 78712, USA; jwpennebaker@gmail.com; 3 Department of Psychology, Smith College, Northampton, MA 01063, USA; bjackson@smith.edu; 4 Program in Culture, Health, and Science, Five College Consortium, Amherst, MA 01002, USA

**Keywords:** interoception, gender, objectification, self-objectification, women’s health

## Abstract

Much of the interoception literature assumes that people can accurately detect their heart rate, stomach contractility, muscle tension, and other biological cues. This is not true. Instead, interoception is an active integrative psychological process where the *feeling* of one’s internal state emerges from physiological signals, contextual cues, and the social and cultural experiences of living in a body. Thinking of interoception this way shifts the focus from measuring accuracy at detecting biological signaling to studying lived experience. One such experience is the widespread objectification of women’s bodies. Living in a body that is chronically evaluated creates a particular form of self-consciousness. Here, we propose that self-objectification redirects attention toward the body, potentially reshaping both the allocation of attention to internal sensations and their interpretation and thereby offering a theoretical account of paradoxes in the interoception literature, such as women’s lower detection accuracy but higher symptom reporting, and mismatches between subjective and physiological reports of menopausal hot flashes. We consider implications for women’s health, including reproductive health, ACL injury risk, and chronic pain. Our framework suggests that “feeling like a woman” reflects an interoceptive experience shaped significantly by objectification, with important consequences for well-being.

## 1. Introduction


*“Man! I Feel Like a Woman!”—Shania Twain*

*“(You Make me Feel Like) A Natural Woman”—Aretha Franklin*


Research on sex and gender differences in interoception has long been framed through a deficit model. In highly controlled lab tasks that ask people to detect autonomic signals such as their heartbeat, women often perform more poorly than men [1]. This has been interpreted as evidence that women are less accurate at detecting internal bodily signals. Yet this sits awkwardly alongside another well-established pattern: women *report* more bodily symptoms, sensations, and emotions than men. To reconcile this, Pennebaker and Roberts [2] suggested that men may draw more heavily on internal cues, while women rely more on contextual information when determining how they feel. Fredrickson and Roberts [3] extended this reasoning proposing that an objectifying cultural milieu which encourages girls’ and women’s vigilance to outer body appearance leaves them less attuned to internal signals. However, both of these frameworks ultimately preserve the assumption of deficiency in women’s interoception.

We consider sex and gender complex constructs, with independent and interactive effects, as articulated in a recent comprehensive review examining why and how brain and behavioral science research can account for sex and gender: “…gender is a complex psychosocial construct that can have profound effects on experiences and, therefore, the brain. In some cases, gender acts independently of sex, and in others, sex and gender interact over the lifespan…p. 6349” [4]. For the purpose of this Special Issue, we focus on women’s and woman-identifying people’s felt experiences of their bodies in an objectifying culture. In this review, we do not attempt to explain sex and gender differences in interoception, nor do we present new data; rather, we argue that experiencing objectification and self-objectification significantly impacts how people experience the signals and symptoms of their own bodies. To do so, we draw on Pennebaker’s [5] earlier work: interoception is not merely the readout of internal bodily signals but a perceptual, constructive process. In framing interoception in this way, we open the door to examining how living in a female body within an objectifying culture reshapes the entire interoceptive process—from attention to bodily cues to interpretations of what those cues mean. What is it like, then, to *feel* like a woman? What about feeling like a *natural* woman? We aim to convince scholars of interoception that the difference between the refrains of Shania Twain’s and Aretha Franklin’s songs is significant to understanding the role of interoception in women’s health. Routinely encountering circumstances that highlight the body’s outer appearance and internalizing an appearance-based perspective on the body (as Shania sings, “short skirts, doin’ it in style”), profoundly alters the entire interoception process, and hence the capacity to feel (as Aretha sings, “so good inside, so alive”) like a natural woman.

## 2. Interoception Is the Feeling of What Happens in the Body

Historically, confusion has arisen in the interoception literature, largely due to differing ways researchers have explicitly or implicitly defined it (see Acknowledgement regarding use of AI tools to organize historical and more recent research in interoception). In the beginning decades of psychology, Hermann von Helmholtz (1852) argued that there was a close link between the intensity of a stimulus (e.g., the loudness of a tone in decibels, the intensity of a shock in volts) and the sensory perception of the stimulus (e.g., perceived loudness or pain) [6]. Later, Fechner (1860) proposed that the perceived magnitude of a sensation reflected the logarithmic function of the physical intensity of the stimulus driving the sensation [7]. In his theory of emotion, William James (1884) argued that perceptions of our visceral bodily signals, finely tuned and specific, served as the basis of our emotions [8].

By the 1970s, interoception research was beginning to apply the same psychophysics models to understand how people detected heart rate, stomach contractility, blood glucose, and other internal states (e.g., [9,10]). Two puzzling effects emerged from these early studies. The first was that men were better at accurately detecting heartbeat [11]—something that went against conventional beliefs that women were more self-aware about internal states. This finding was hard to square with studies showing that women *report* more bodily distress and more numerous, more intense, and more frequent somatic symptoms than men (e.g., [12,13]). The second consistent finding was that “accuracy” at detecting internal states using highly controlled lab tests was generally very poor, with most people performing at levels close to chance. In studies with multiple trials for each participant, people accurately detected their own heartbeat around 55% of the time, where 50% was chance. Indeed, males were slightly better than females (perhaps 57% vs. 53%), but these controlled studies indicated that nobody was very good. Similarly low rates of accuracy in highly controlled lab settings have been found with other systems (e.g., breathing rate, finger temperature, blood pressure [14,15]).

Ironically, almost two decades earlier, the psychophysical approach to visual perception started to be challenged by James Gibson (e.g., [16]), who argued that our perception of all sensory experiences reflected a broader perceptual system. For example, in our everyday experience, we may perceive that our heart is racing because we see a horrible scene, we win an unexpected prize, or we step into a very cold shower. Several studies have demonstrated that these outside-of-the-body cues contribute far more to our perceived heartbeat than our actual heartbeat. For example, when assessing perceptions of heart rate under various conditions (loud noise bursts, placing a hand in freezing water), Pennebaker and Epstein [14] found that people’s estimates tended to reflect their beliefs rather than reality. People report, for example, that their heart rate goes up when in cold water. In fact, it goes down. As well, people report that ice water *feels* colder when they see their submerged hand in a mirror than when no mirror is available [5,17]. In other words, our internal biological cues provide a very weak signal that can be swamped by far more salient cues outside the body.

This more ecologically valid framework provided the foundation for Pennebaker’s book *The Psychology of Physical Symptoms* [5], which anticipated a much broader conceptualization of interoception processes that has emerged in research of the following decades [18]. Although the field has progressed substantially since then, several problematic assumptions identified by Pennebaker and his colleagues [17] continue to influence interoception research [18]. Among these are the assumptions that: (a) interoception can be operationalized as a detection ability, best measured in laboratory settings that attempt to exclude external environmental “confounds,” and (b) the ability to detect any given autonomic response measure (e.g., heart rate, blood pressure, stomach contractility) is behaviorally relevant in predicting more adaptive or healthy outcomes for humans. Both assumptions are false. In the real world, people use whatever information is available to them to “know” how they feel. This means controlled laboratory studies are missing much of the data people use to define their internal states in everyday life.

Instead, when defined as the *perceptual process* of representing one’s internal state (cf., [19]), interoception is highly influenced, not confounded, by attention to and conceptualizations of inputs from both inside and outside the body. In our phenomenological account, biological signals are events (e.g., heart rate) that are distinguishable from sensations, which are feelings (e.g., “I feel my heart beating”). Rather than readouts of physical events, we treat sensations as experiences—as the feeling of what happens in the body. Consistent with Pennebaker’s [5,17] observation that the ability to detect a particular physiological event has limited behavioral relevance, we propose that behavior is shaped not by the physical signals themselves, or by our ability to detect them, but by the perceived bodily state, which emerges from the integration of multiple internal and external sensory inputs.

## 3. The “His and Hers” Model of Gender and Interoception

In the early 1990s, two of us relied on the Gibsonian ecological perception framing to interpret the sex differences in interoceptive accuracy noted at the time [2,20]. Basically, highly controlled lab studies tended to demonstrate that men were more accurate at detecting internal signals such as heartbeat and blood glucose manipulations. However, outside the lab, in naturalistic diary or experience sampling studies, no sex differences were evident in the accuracy of estimates of blood pressure, blood glucose, heart rhythm, or heart rate (e.g., [21,22]). We reasoned, then, that men and women may rely on slightly different types of cues in determining feeling-states, with men relying more on inside-body cues and women more on situational/contextual cues.

We offered three potential explanatory mechanisms for this difference: biological (e.g., hemispheric lateralization); social dominance (those with lower status in society, such as women, are more vigilant to the social environment for navigating social acceptability and safety); and gendered socialization of beliefs about the body (women’s reproductive and sexual bodies are framed as more shameful, disruptive, and unpredictable than men’s). Ultimately, we proposed “His-and-Hers” theories of emotion, with men’s interoceptive style aligning more with William James’s (1884) theory of emotions as perceptions of bodily changes [8], and women’s approach reflecting cognitive appraisal theories of emotion such as Magda Arnold’s (1960) [23], which emphasize contextual meaning-making in determining felt-states [20].

In the ensuing 30 years, further research has examined sex and gender differences in interoception, but the emerging picture is hardly clarifying. A meta-analysis of 93 studies of interoceptive accuracy across cardiac, respiratory and gastric domains concluded that males demonstrate greater accuracy in cardiac, but not gastric signal-detection tasks, while findings on respiratory tasks were mixed [24]. Studies of heartbeat detection using newer technologies, such as the phase adjustment task, found that females outperformed males in accuracy but showed lower confidence in their judgments, supporting possible differences in the meta-cognitive elements, likely the product of gender socialization, of interoceptive awareness [25]. Similarly, Grabauskaitè et al. [26] used the Multidimensional Assessment of Interoceptive Awareness (MAIA) self-report scale [27], which measures subjective awareness of various facets of interoception, as well as a heartbeat counting task, to compare healthy male and female participants. They found that while males were more accurate in consciously detecting heartbeats, females reported noticing bodily sensations (including heartbeat, breathing, muscle tension) more often, showed better understanding of the relationship between bodily sensations and emotional states (e.g., muscle tension goes with stress), and experienced more emotional distress from sensations of pain or discomfort.

Complicating this picture even further are studies of menopausal hot flash experiences, with only women participants. Up to 75% of women in the United States report experiencing hot flashes during the menopausal transition, which arise from activation of the heat-dissipation response, likely triggered by hypothalamic mechanisms in the context of declining estrogen [28,29]. Hot flashes are measured both via subjective reports and objective physiological measures (e.g., heart rate, blood flow, temperature, and especially sternal skin conductance). Interestingly, and in contrast to women’s lower interoceptive “accuracy” in heartbeat detection tasks when measured in the lab, subjective reports of hot flashes can approach 100% concordance with objective physiological measures in the laboratory. However, in ambulatory studies of hot flash reporting, this subjective-objective concordance is significantly lower, with many hot flashes that register on sternal skin indicators not reported, and subjective reports of hot flashes not registering on physiological indicators [30].

On reflection, our original “his and hers” theorizing, and much of the ensuing research hunt for meaningful gender differences in interoception, have fallen under the spell of the same faulty assumptions Pennebaker himself had warned against. Namely, they assume that interoceptive accuracy is a measurable skill, and that differences in this skill predict more or less adaptive behavioral outcomes [17,18]. Research increasingly fails to support that interoception is akin to a generalizable *ability* to detect objective phenomena (e.g., [31]). Furthermore, considering how poorly all people perform on interoception accuracy tasks, the mixed results of these studies make sense. The gender difference studies account for a tiny amount of variance in tasks in which both genders are barely able to perform above chance, and they are juxtaposed with far larger gender differences in self-reported awareness of bodily sensations. And in hot flash studies of only women, interoceptive accuracy in and out of the lab is flipped: with *greater* so-called accuracy in the lab than out in the world. Finally, surely these small accuracy differences get us no closer to appreciating whether and how gender might be meaningfully, clinically relevant to interoception.

## 4. Feelings and Emotions as Phenomenological Constructions

New psychological theories of emotion have also been developed in the years since our “his and hers emotions” proposal. These models show that determining how we feel is a construction process, an idea aligned with Gibson (1966) [16]. Lindquist’s (2013) theory argues that emotions such as fear, disgust, joy, anger, or pride emerge in consciousness out of two ingredients: core affect (arousal level and valence) and conceptualization (interpretation, categorization) [32]. Core affect is grounded in internal physiological signals. For example, a racing heart and stomach tension might contribute to a core affective state of high arousal and negative valence. Importantly, core affect is not categorical, but rather continuous. Conceptualization is an ongoing process of making sense of inside-body cues and cues from environments we find ourselves in. It is grounded in prior knowledge, experience, socialization, and language. For example, one person, given their context, history, and language, might parse the ongoing core affective stream as “excitement.” Another person in a different context, or with different prior knowledge and interpretive frames, might experience “anxiety.” That is, experiencing sensations in the body may provide a clue that something is going on in any given here-and-now context. Conceptualization finds an emotion label for what the feeling in this context means for a given person.

Indeed, research supports a three-step neural model for perception of the body and emotions, including discrete bodily sensations, whole-body patterns, and emotion concepts [33]. Extending this framework, Feldman Barrett and colleagues [34] have offered a broader theory of constructed emotion in which interoception plays a central organizing role. In this account, the brain actively constructs emotional experience by integrating incoming sensory inputs with prior experience and learned concepts. Interoceptive signals contribute to “core affect,” but these signals are always interpreted through predictive models shaped by culture, language, and personal history.

This reconceptualization aligns closely with advances in computational neuroscience, particularly predictive coding models (e.g., [35,36,37]), which propose that the brain generates predictions about incoming sensory input and updates those predictions based on prediction error. Applied to interoception, this means that what we feel from the body is shaped as much by physiological signals as by expectations, reflecting the brain’s best guess about the causes of bodily sensations in context.

Constructed emotion models provide an important step toward resolving a paradox raised by our original “his and hers” model of emotion. If inside body cues inform core affect, and males are more accurate at detecting such cues, how can we explain women’s advantage over men in the processing and recognition of their own and others’ emotions (e.g., [38])? Prentice et al. [24] extended and updated our early postulating by proposing that language socialization provides different conceptualization frames for men and women to make sense of core affect. They point to evidence that caregivers and culture socialize boys and girls into different internal-state language, with boys receiving more physiological labels and girls more emotional labels. Over the course of development, then, men make more physiological interpretations and women more emotional interpretations of the ambiguous bodily signals that inform the stream of core affect. As an example, when experiencing a racing heartbeat, males may be more likely to use the conceptualization “I drank too much coffee” while females may be more likely to conceptualize this internal state as “I am anxious” ([24], p. 3).

Predictive coding models, together with gendered language socialization, offer a coherent account of seemingly incompatible findings regarding gender differences in interoceptive and emotional abilities. Rather than locating these differences in superior or inferior “detection” of bodily signals, this framework highlights how gender shapes the predictive models and conceptual frames brought to bear in answering the question, “How do I feel?” However, even this framing preserves the assumption that there exists a stable, objectively detectable bodily signal awaiting accurate registration. Here again, we see echoes of the concerns raised by Pennebaker [18]. Our position presses further. From a phenomenological standpoint, interoception is not best understood as signal detection, but rather as an embodied perceptual process embedded in the lived experiences of human beings beyond the laboratory [39].

## 5. Objectification Theory: Key to the Phenomenology of Living in a Female Body

When we consider interoception as an *entirely perceptual, constructivist process* of representing one’s internal state—from attention to bodily sensations all the way to appraisals—then it is important to consider the experience of *living in a female body*. Objectification theory [3] provides a model for doing so. Objectification is a phenomenon wherein human beings are reduced to mere objects, things, or tools (e.g., [40,41]), and can occur in several arenas, ranging from relatively harmless to extremely harmful. For example, medical students or pathologists objectify the human cadavers in their labs to perform their duties without the emotion that comes from imbuing the human body with personhood [42]. Sexual objectification has been described by feminist philosophers and historians as the treatment of women as sexual things or commodities, illustrated in cultural depictions from Renaissance painting to advertising to pornography [43,44,45]. Fredrickson and Roberts (1997) offered objectification theory to the behavioral and social sciences as a theoretical perspective to guide empirical research on the psychology of sexual objectification [3].

Objectification theory proposed that sexual objectification occurs both culturally and interpersonally. With globalization, ever more widespread depictions of the sanitized, deodorized, denuded, sexually attractive youthful female body appear across media and advertising domains. Interpersonally, girls’ and women’s bodies are also treated as *things*, along a continuum from sexualized evaluation (e.g., ogling, cat calling, commenting on social media) to more extreme forms such as assault or trafficking. Indeed, in Western culture, women encounter an objectifying event on a nearly daily basis [46,47,48,49]. In this milieu, girls and women are socialized to become their own first surveyors, internalizing and even becoming preoccupied with this appearance-dominant perspective on their bodies, essentially to sexually objectify themselves. This *self-objectification* leads to a form of self-consciousness theorized to fragment consciousness, as cognitive resources are taken up by attending to and monitoring the body’s outward appearance [3].

Self-objectification can be measured as a *trait*, with some individuals adopting an appearance-over-competence perspective on themselves more than others across situations and the lifespan. Several trait measures of self-objectification have been developed; these typically ask about the extent to which individuals prioritize their body’s looks over its functionality or engage in habitual body self-surveillance and appearance-enhancing behaviors (e.g., [50,51]). Generally, women score higher than men on these scales, but within gender samples show relatively normal distributions. Self-objectification can also occur as a *state*, when present-moment contexts place a focus on the body’s appearance over its competence. These are in a reciprocal relationship; the lifelong consequences of the chronic induction of this state among girls and women include negative emotions such as shame and depression, which result from falling short of meeting cultural ideals. These experiences interact with self-schema such that with repeated exposures to objectifying circumstances, self-focused rumination on negative emotions acts as a “glue” to solidify states of self-objectification into chronic, trait self-objectification [52,53].

Whether as a trait or a state, self-objectification is not only associated with myriad negative emotional, but also cognitive and behavioral consequences (see [54]). For example, one study showed that girls higher in trait self-objectification threw a softball less effectively (ergonomically poorer preparatory arm backswing and humerus action), illustrating that “throwing like a girl” may be explained in part by the splitting of attention between how the body looks while performing an action and the movements required for the action [55]. In another study, trying on a swimsuit (as compared to a sweater) led to impaired math performance among female but not male participants [56], presumably because such a state of self-objectification directed women’s attentional resources to the body’s appearance, which limited available cognitive resources for other tasks.

Importantly, subsequent studies suggest the negative impacts of states of self-objectification may be relevant for all people. For example, Hebl et al. (2004) used the swimsuit-sweater paradigm with one modification—male participants tried on revealing Speedos instead of swim trunks—and found that this increased state self-objectification for *both* men and women [57]. Similarly, being the target of an evaluative gaze or viewing appearance-ideal (i.e., muscular) male bodies also increases self-objectification and appearance anxiety in men [58,59]. One study of transmen and transwomen showed that experiencing microaggressions regarding their gender presentation increased self-objectification and, in turn, shame [60]. Notably, however, studies of state self-objectification in non-female samples generally show smaller effect sizes and fewer downstream consequences, such as impaired cognitive performance. Therefore, because girls and women experience more states of self-objectification in a sexually objectifying world (they are more likely to wear revealing clothing to meet sexualized standards of ideal appearance, or to be cat-called while walking, for example), they are more likely to experience the attentional and emotional consequences of state self-objectification more often, but boys, men and trans folks can be induced into such a state as well.

Patterns of objectification and trait self-objectification, along with their attendant consequences for health and well-being, vary by sexual orientation [61], age [62], able-bodiedness [63], gender identity [64], and ethnicity [65]. As well, though research consistently demonstrates higher objectifying treatment and greater trait self-objectification in female populations than male, boys and men may experience objectification during interactions with others [66]. Men who self-objectify also report more body shame and disordered eating [67]. Further, gay men, who exist in a subculture that emphasizes physical appearance, report more self-objectification and related negative consequences than heterosexual men [68]. Recent research shows that the highly visual nature of social media platforms like Instagram or TikTok contributes significantly to body image concerns among adolescents and young adults. Because the appearance of the body is so heavily emphasized, girls and women remain at higher risk of the consequences of objectification and self-objectification both online and offline. Crucially, however, research indicates that boys and young men are increasingly vulnerable to these same experiences (e.g., [69]).

Fredrickson and Roberts (1997) [3] originally theorized that self-objectification’s vigilance to outer body appearance also reduces awareness of internal physiological cues. Although over a thousand studies support the theory’s psychological and behavioral predictions, the interoception hypothesis remains relatively less explored. Studies have found that self-reported interoceptive awareness mediates the relationship between self-objectification and disordered eating [70,71]. Notably, one study found that higher trait self-objectification predicted poorer heartbeat tracking accuracy among women but was *un*related to subjective interoceptive beliefs [72]. This disjunction replicates early findings [14] that accuracy and beliefs about interoception were independent. As with studies of menopausal hot flashes, where many physiologic hot flashes go unnoticed, and sometimes women report hot flashes that do not appear on psychophysiological monitors [30], here some participants believed they were very in tune with their bodies’ internal signals but were not accurate heartbeat trackers, while others were more accurate at detecting heartbeats but did not describe themselves as particularly “interoceptively aware”.

## 6. Man! Feeling Like a (n Objectified) Woman

The disconnect between objective accuracy and subjective reports observed among women in these studies requires a revision of Fredrickson and Roberts’s (1997) original theorizing [3]. The influence of self-objectification on interoception does not merely result in a simple internal cue detection “deficit” or even reduction in self-reported awareness. Instead, when interoception is defined as the constructive perceptual process that represents the feeling of what happens in the body, then both trait self-objectification and state self-objectification fundamentally alter this psychological process. Circumstances that spotlight the body’s appearance or vulnerability (e.g., real-life and digital environments, clothing, advertising) have profound implications for the entire interoceptive continuum: from attention to and awareness of inside-body sensations to appraisals or conceptualizations used to answer the question, “How do I feel?” Therefore, objectification and self-objectification produce a unique, socially conditioned form of internal experience: an interoceptive “*feeling* like a woman”.

### 6.1. Objectified Interoception: Attentional Processes

Objectification and self-objectification influence the attention-relevant elements of interoception in at least three distinct ways. The first involves inattentional blindness via attentional resource depletion. The cognitive load associated with appearance self-monitoring exhausts the attentional resources necessary for encoding subtle physiological cues. Second, self-objectification steers the attentional spotlight selectively. Attention may be actively diverted away from ambiguous internal sensations and redirected toward more externally relevant appearance cues, sometimes even devolving into a ruminative focus on the body’s appearance “failures.” Finally, self-objectification may result in sensory adaptation by way of habitual body-altering practices. Repeated engagement with such practices may lead to diminishing subjective awareness of bodily sensations.

*Inattentional Blindness.* Inattentional blindness is a perceptual failure in which people do not perceive some stimuli because their attention is engaged elsewhere, and most often occurs under conditions of cognitive demand [73]. Some studies have confirmed that one way self-objectification impacts interoception is via the same attentional resource depletion mechanism that explains reductions in cognitive processing (e.g., math tests) or physical movement (e.g., throwing a softball). A few correlational studies show that women higher in trait self-objectification report lower “body awareness” as measured by noticing how foods impact the body or feeling a fever coming on [74] and lower awareness of hunger cues [70]. The body surveillance associated with high trait self-objectification has been shown to predict disordered eating [75]. And in the swimsuit sweater study manipulating state self-objectification, women who had tried on a swimsuit and experienced greater body shame subsequently ate significantly less in a seemingly unrelated taste test than those who tried on the sweater, while the clothing manipulation did not affect men’s eating [56].

The attentional resource depletion mechanism for self-objectification’s impact on interoception may also explain findings related to sexual arousal and responsiveness. For example, sex researchers find that “spectatoring” (monitoring the body’s appearance while engaging in sexual activity) disrupts sexual arousal (e.g., [76]). Furthermore, trait self-objectification predicted reduced attention to internal sexual sensations [77], and the body surveillance characteristic of self-objectification is associated with reduced attention to sensations of genital arousal and reduced sexual responsiveness [78].

*Selective Attention.* Selective attention is the ability to prioritize and process certain information while inhibiting or ignoring other inputs [79]. This may be a second mechanism by which objectification and self-objectification impact interoception. Studies show girls and women engage in high rates of dieting and restrained eating starting in early adolescence, in efforts to achieve or maintain the slim, attractive body ideal required of them in a sexually objectifying culture [80,81]. Importantly, dieting and restrained eating require selectively ignoring and even actively down-regulating hunger cues [82,83], and this likely contributes to the fact that such habits predict later eating pathology [84]. For example, restrained eaters in a water load task showed blunted reports of fullness compared to non-dieters [85], and lower accuracy on heartbeat detection tasks [86]. An experimental study of satiety in food consumption manipulated state self-objectification by heightening appearance focus among women participants with a brief mirror exposure or viewing advertisements of models. This led them to rely less on satiety signals in their eating behavior, operationalized by them eating *more* snacks after a filling lunch [87].

While the habitual restrained eating characteristic of self-objectification requires selectively *not* attending to hunger and satiety cues, it may simultaneously predict selectively attending *to* sensations which may signal “fatness.” Indeed studies show that habitual dieters and restrained eaters selectively attend to somatosensory cues *on* the body, especially how clothing fits, how large the body feels from the outside (for example, scanning for the feel of “fat rolls” when sitting, touching the stomach or thighs), and how the body occupies space [88,89]. This is part of a broader pattern of heightened body surveillance and body checking associated with self-objectification. As such, selective attention trained toward appearance-related cues sets the stage for rumination—persistent, repetitive focus on perceived bodily flaws or failures (e.g., [90]) and this ruminative thinking, in turn, helps sustain and amplify self-objectification [52].

*Adaptation.* Attentional systems recalibrate, depending on what people repeatedly monitor or ignore (e.g., [91]). This is a third way in which objectification and self-objectification may impact interoception. Women account for approximately 84% of aesthetic plastic surgeries and procedures worldwide [92]. Studies show that these women, and increasingly younger and younger girls, actively normalize or dismiss postoperative and posttreatment pain from aesthetic procedures as necessary for beauty or self-confidence (e.g., [93,94]). Sometimes such procedures have dire consequences. For example, in *The Manicurist’s Daughter: A Memoir* Susan Lieu (2024) traces how the pressures faced by women in her Vietnamese refugee community, combined with a largely unaccountable cosmetic surgery industry, shaped her family’s life and led to her mother’s death after a botched procedure [95].

Adaptation to discomfort from objectification is normalized even in less extreme, more daily life. Higher trait self-objectification is associated with normalizing painful or potentially injurious “personal grooming” such as pubic hair removal [96] or skin bleaching [97]. In a field study titled, “When looking ‘hot’ means not feeling cold,” experimenters approached women outside nightclubs on cold nights, assessed trait self-objectification and noted skin exposure (many were wearing “short skirts, doin’ it in style” as Shania sings). Participants were asked to report how cold they felt. We found that women low in self-objectification showed a positive, intuitive, relationship between skin exposure and perceptions of coldness, but high self-objectifying women did not report feeling colder when more skin was exposed from wearing less clothing [98].

### 6.2. Objectified Interoception: Conceptualization Processes

Objectification and self-objectification also influence the interoceptive perception process by providing “feeling like a woman” conceptualization frames. Just as caregivers socialize boys and girls into differing internal-state language [24], they also shape children’s understanding of their bodies through the appearance-based language they model and direct toward them. A number of studies have demonstrated that mothers’ appearance self-criticism and “fat talk” about their own bodies provides daughters with a linguistic and conceptual template that prioritizes how the body looks over how it feels. These maternal patterns of speech predict daughters’ heightened body surveillance, diminished body esteem, and increased internalization of appearance ideals [99,100]. Importantly, recent evidence suggests that parental attention *to* their children’s physical appearance—particularly from fathers—is associated with greater body shame in both girls and boys [101]. Thus, parental language, combined with the commentary provided by peers, and increasingly more importantly, social media [102], likely develops gendered and self-objectifying conceptualization frames for the whole interoception process, from how to interpret bodily sensations, to whether the inside or the outside appearance of the body matters more in answering the question, “How do I feel?”

To illustrate, research shows that the benefits of “power posing” [103]—which involves both interoception and proprioception—are not as straightforward as originally predicted. In one study, while upright posture reliably elicited feelings of pride and confidence in men, women did not experience the same and, in fact, showed greater pride in a slumped posture [104]. Huang et al. [105] showed that expansive posture increased men’s sense of power, risk-taking, and performance, but produced weaker or even negative effects for women, who were sometimes penalized with social backlash when adopting the same postures.

One experiment tested whether these differing effects could be explained by self-objectification. Women participants were assigned to high or low status roles while wearing a tight-fitting tank top or a loose sweatshirt. Expansive postures improved performance for women assigned high-status roles, but in conditions of low status or heightened awareness of their bodies’ appearance, the same postures increased negative affect [106]. As we have argued, self-objectification provides a conceptualization frame that prioritizes the outside appearance of the body in answering the question, “How do I feel?” In this case, self-objectification, heightening awareness of women’s appearance and, particularly, their breasts (objects of sexualization in Western culture), may take the power out of power-posing.

## 7. Discussion

### 7.1. Feeling Like a Woman in the Doctor’s Office

What are some practical implications of our theoretical framework for interoception’s role in women’s health? In a culture of objectification and self-objectification, appearance focus is often driven by fear, anxiety, and shame, which perpetuate further self-objectification. Focusing on one’s looks and the downstream implications for interoception can be costly to health, even deadly. Here, we offer two examples where the attentional and conceptualizing mechanisms of self-objectifying interoception are important to consider: reproductive health and injury, and chronic pain.

*Reproductive Health.* Women’s reproductive functions activate cultural anxieties about people’s biology, animality and mortality [107,108]. Objectification and self-objectification offer a psychological means of distancing themselves from these reminders by prioritizing appearance over the messy realities of menstruation, menopause, pregnancy and lactation. For example, menstruation stigma teaches women to treat their cycles as contaminants requiring secrecy [109]. Even an accidental disclosure, such as dropping a tampon, leads others to like and respect them less [110]. Higher trait self-objectification in women predicts more emotions such as disgust and shame toward menstruation and even opting for menstrual suppression to eliminate periods altogether (e.g., [111,112]). Similar norms govern pregnancy and lactation. Pregnant and postpartum women higher in self-objectification show more body shame, more concerns about the negative impact of breastfeeding on their bodies and sexuality, barriers to breastfeeding, and fear of childbirth [113]. Thus, objectification and self-objectification support hiding, suppressing and silently managing menstruation, menopause, pregnancy and lactation, so it is no wonder women higher in self-objectification are less likely to seek preventative sexual and reproductive healthcare [114].

Indeed, reproductive health-related conditions are underreported and underdiagnosed, despite women reporting more symptoms and seeking more healthcare services generally than men. We predict that self-objectification cultivates the very attentional biases we have argued impact interoception: inattentional blindness and selective attention away from bodily sensations, as well as long-term adaptation to discomfort and pain. Consistent with this idea, a 2021 study showed that only 55% of women receive preventative gynecologic care [115]. A study of over 3000 women from three countries found that 90% of dysmenorrhea-affected women did not seek medical attention or advice [116]. Endometriosis affects an estimated 10% of reproductive-age women worldwide, and even higher percentages (up to 50%) of women with chronic pelvic pain or infertility [117,118,119]. However, the symptoms—severe cramps, heavy bleeding, bowel or bladder pain, pain during sex—are often dismissed by both patients and doctors as normal, or even “in one’s head,” leading to an average delay of 7 to 10 *years* between symptom onset and diagnosis [120]. It may be that interoceptive disruptions, along with cultural shaming around reproductive health, undermine early detection and reporting of symptoms such as abnormal bleeding, mastitis, or pregnancy complications.

Self-objectification may also provide an explanation for the disjuncture between subjective and objective reports of hot flashes during menopause noted earlier, which appear to show greater correspondence in the lab than in the field. Trait self-objectification predicts poorer attitudes toward menopause, reflected in selective attention to perceived negative appearance-related effects such as decreased skin elasticity, wrinkles, or weight gain [121]. In an objectifying culture, many women’s attention during menopause may be more attuned to noticing feelings of sweating and flushed skin while they are out in the world interacting with others, given that these symptoms would be visible markers of their aging bodies. Indeed, hot flashes produce social embarrassment, shame, and anxiety for many women when experienced in public, and this ruminative selective attentional bias can act as an amplifier of hot flashes [122].

These salient subjective experiences would be the ones reported to doctors. However, they may not correspond to physiological vasomotor changes. In one diary study, where women tracked hot flashes at the beginning and end of each day while wearing monitors, women actually *under*estimated the number of vasomotor events they experienced compared to those registered by the physiologic monitoring. Importantly, physiologically assessed hot flashes, not subjectively reported ones, predicted vascular dysfunction that could have significant heart-disease consequences [123]. This suggests that relying only on self-report is likely to lead to under-recognition of risk.

*Injury and Chronic Pain*. Women sustain anterior cruciate ligament (ACL) injuries at rates two to eight times higher than men, with long-term consequences such as early osteoarthritis (e.g., [124]). These disparities are often attributed to anatomical (e.g., pelvic width–femur length ratios) and physiological, hormonal factors (e.g., estrogen effects), which provide both the self- and medical diagnostic conceptualization frame for understanding gender differences in ACL injury. However, a growing body of work suggests that this is a misattribution, with potentially insidious consequences. Gender socialization in an objectifying culture includes self-objectifying movement norms that foster chronic self-monitoring and outwardly focused attention.

As we have shown, self-objectification predicts more restricted movement, with a focus on one body segment versus the entire body, limiting full use of the body’s spatial potentialities [55], accounting for phrases such as “throwing like a girl” or “sitting like a lady.” This has been theorized to produce habitual postural and kinematic patterns that increase ACL vulnerability, and indeed one study showed that higher trait self-objectification predicted (1) greater side-to-side hip movement on the non-dominant leg during a single-leg stand-to-sit, (2) less hip movement on the dominant leg during a drop vertical jump, and (3) less rotational knee movement on the non-dominant leg during stand-to-sit. These movement patterns align with what has been described as the “position of no return” in non-contact ACL injuries [125]. From our perspective, adapting to the neuromuscular demands of “sitting like a lady” may lead to inattentional blindness toward the interoceptive and proprioceptive cues that are essential for safe landing and joint-stabilizing movements. In this way, self-objectification provides a mechanism by which cultural expectations literally “get under the skin,” shaping neuromuscular control and contributing to women’s elevated ACL injury risk.

When not treated, physical injury can lead to chronic pain that persists long after the acute tissue damage heals. Women consistently show higher percentages of chronic pain and report feeling pain more intensely and for longer durations than men in the United States [126]. In recent years, researchers and practitioners have identified numerous examples of how origins of pain are not limited to tissue damage or nerve injury. The latest pain science aligns with our perspective on interoception, showing that nociplastic pain is a perceptual, contextually constructed experience generated by altered central nervous system processing rather than ongoing tissue damage or nerve injury [127]. To illustrate, chronic stress activation and impaired emotion regulation due to fear and anxiety have been argued to be a mechanism by which societal inequality and discrimination alter brain processing to cause pain [128]. For women in an objectifying culture, chronic exposure to objectification and self-objectification likely also dysregulates stress-response systems in ways that heighten physiological vulnerability to pain. Further, because these experiences cultivate ruminative shame, anxiety, and vigilance about the body’s appearance, pain is likely amplified.

Support for this perspective on pain comes from a randomized clinical trial evaluating Pain Reprocessing Therapy (PRT), a psychological treatment designed to reduce chronic pain by retraining how the brain interprets pain signals [129]. Close to 70% of the PRT group were pain-free or nearly pain-free post treatment, compared to only 20% of placebo and 10% of usual care patients. Neuroimaging showed reduced activation in pain-related brain regions, including the anterior midcingulate cortex and anterior insula, after PRT, consistent with changes in pain processing. Relevant to our revised model of interoception, if women’s greater chronic pain is in part caused by the interoceptive disruptions of objectification and self-objectification, then so, too, can it be *reconceptualized* and effectively treated by redirecting selective attention, short-circuiting the fear response that likely engenders inattentional blindness, and re-framing cognitive conceptualizations of what pain sensations mean.

### 7.2. Future Directions: Recommendations for Practitioners

We have argued that interoception is constructed by interactions among a range of attentional and conceptualization or meaning-making forces. This suggests there are multiple potential levers for deliberately changing interoception. Body objectification is argued by feminist psychologists to be a human rights violation, with the ensuing self-objectification and its consequences characterized as *psychological cliterodectomy* [130]. Preoccupied with their own body projects while also doing the disproportionate amount of unpaid care labor [131,132], “objects don’t object” [133]. This perpetuates a system of gender inequality, harming not only women themselves but others alike. In the face of these societal constraints, what is to be done to support women and women-identified people to feel, as Aretha sings, if not “so good” then at least better “inside”? “So alive?” In fact, a lot.

As we have shown, self-objectification’s impact on interoception shapes a wide range of health behaviors and resulting symptoms and conditions. An important consequence is that self-objectification may be a *final common pathway* in understanding women’s embodied experience. Just as many health practitioners screen for mental health symptoms, we recommend incorporating brief self-objectification questionnaires as part of general intake to assess trait self-objectification (cf. [125]). High self-objectifiers may benefit from treatment plan discussions that acknowledge—without judgment—interoception and symptom tracking challenges that could arise, particularly if illness or injury impacts the body’s appearance.

Although clinical settings are often sterile, waiting rooms and exam rooms can be designed not only with practitioners but also with users in mind [134]. Such settings may induce either self-objectification or self-agency for a patient. This could be through the placement of furniture, whether and how patients are asked to change clothing, and the arrangement of doors and curtains, that maximize or minimize potential exposure. Making such changes will require not only skills-building of individual practitioners, but also health systems that offer effective training for practitioners in the therapeutic power of interaction spaces that afford deeper listening. These are not only architectural, but also structural support (e.g., time in appointments, staffing coverage) and financial incentives (e.g., insurance reimbursements).

Though there are important structural improvements needed in healthcare provision and delivery, there are meaningful incremental changes practitioners can make in the shorter term. On the individual level, it is helpful for patients to break the cycle of body-focused rumination and ensuing shame as a key first step to “loosen the glue” of self-objectification in constraining interoceptive processing. Health care professionals must support patients to broaden and build their range of internal psychological resources. This is through creating internal perceptions of safety, the groundwork for warm, positive emotions, which can build physical health (e.g., [135]). A critical antidote to objectification is humanization. Our work suggests that health care practitioners should consider two broad levers in the clinical encounter: (1) ways for practitioners to humanize their patients, (2) ways to help patients humanize themselves. Growing evidence suggests interoceptive interventions can optimize behavioral treatment outcomes for a variety of distressing conditions [39]. We urge intervention research to determine whether our recommendations for reducing objectification and self-objectification do, in fact, improve patients’ clinically relevant interoception and symptom perception self-efficacy.

*Humanizing Patients.* When patients—women and others alike—are carefully listened to, their experiences are validated, and painful emotions accompanying the experience are well-enough processed, symptoms and pain can often resolve [136,137]. At first glance, it may seem most equitable to “not see gender.” However, as long as people’s experiences are shaped by social arrangements and processes that target and treat bodies differently by gender, it is more humanizing to notice and acknowledge, when therapeutic, the experiences of patients. We might argue that gender itself is an interoceptive process by which all individuals construct identity from their assigned sex; therefore, we urge practitioners *to* pay attention to gender and to be curious about how objectification and self-objectification might be relevant to patients’ experience.

In doing so, important objectification-relevant processes may illuminate clinically important differences among women, and among those who do not live a life-course trajectory as cisgender. One study of transmen showed a negative relationship between transgender identity congruence (feeling like one’s body appearance matches one’s sense of identity) and body appearance surveillance, with greater congruence predicting less body surveillance [138]. This suggests that more congruently masculine individuals have less self-objectification. Another study found that, unlike ciswomen, transwomen experienced sexual objectification as a positive sign of being read as feminine [139], suggesting that being objectified as a woman may *increase* transwomen’s identity congruence and thus decrease the body surveillance typically characteristic of ciswomen’s self-objectification. These studies underscore both the need for more research on trans- and gender non-binary people’s experiences of objectification, self-objectification and interoception, as well as the potential benefits of practitioners’ curiosity about their patients’ experiences of their gendered bodies. It is also critical to keep in mind that gendered objectification intersects with other social categories that mark bodies, such as racialization, age, socioeconomic status, immigration status, religion, geography, (dis)ability, and skin tone, among others.

*Patients Humanizing Themselves.* The more that practitioners humanize patients in their care by viewing and treating them as whole people in clinical encounters, the more that patients will continue humanizing themselves. Practitioners can coach their clients once they leave the clinic to counter the immense sociocultural pressures toward self-objectification. *Cultivating mindful self-awareness* is a counterpoint to inattentive blindness, selective attention, and adaptation. Instead, developing mindful self-awareness is the process of deliberately recalibrating one’s attentional focus from how one’s body looks toward one’s internal perceptions without judgment. Recent research validates that mindfulness can lessen self-objectification [140]. This can be helped by *finding agency in bodily lived experiences*. Enjoyable physical activity of one’s choosing is one of the most powerful and empirically supported ways for females across the lifespan to promote the functioning of physiological systems while also countering the development of trait self-objectification (cf. [141]). With the ubiquity of objectification, particularly with the rise of visual culture in the 21st century, using social media has been associated with greater self-objectification and, in turn, negative mood [142] among other body-relevant outcomes [143]. In this milieu, learning to *practice self-compassion* as one consciously works to interrupt self-objectification is necessary. Finding mindful self-awareness and bodily agency may not come easily after a lifetime of socialization otherwise. Self-compassion supports patients to tolerate the messy unlearning of self-objectification as a default stance (cf. [143]).

## 8. Conclusions

Objectification and self-objectification can cultivate interoceptive feelings in the ways Shania Twain sings about. We would like to close with the promise of women and female-identifying folks feeling “so alive,” like the natural woman Aretha Franklin sings of. This form of *feeling* “natural” is not about anything fixed or biologically predetermined. Instead, in the context of interoception as a wholly constructed perceptual process, Aretha’s lyric points to a more human, open, and genuine experience of inhabiting one’s body: one in which internal sensations can be encountered without the distorting lens of objectification. When women are freed from cultural demands to monitor how their bodies appear, they can engage more fully with how their bodies feel. In this sense, to feel like a “natural woman” is not to return to some essential truth, but to inhabit a mode of interoception that is spacious, enlivened, and fundamentally human.

## Data Availability

No new data were created for this review paper.
